# Electrochemical Biosensors Based on S-Layer Proteins

**DOI:** 10.3390/s20061721

**Published:** 2020-03-19

**Authors:** Samar Damiati, Bernhard Schuster

**Affiliations:** 1 Department of Biochemistry, Faculty of Science, King Abdulaziz University (KAU), Jeddah 21589, Saudi Arabia; sdamiati@kau.edu.sa; 2 Institute for Synthetic Bioarchitectures, Department of NanoBiotechnology, BOKU - University of Natural Resources and Life Sciences, Vienna, Muthgasse 11, 1190 Vienna, Austria; 3 Current address: Division of Nanobiotechnology, Department of Protein Science, Science for Life Laboratory, School of Engineering Sciences in Chemistry, Biotechnology and Health, KTH Royal Institute of Technology, 171 21 Solna, Stockholm, Sweden

**Keywords:** S-layer proteins, biosensor, biocompatible layer, self-assembly, bioinspired material

## Abstract

Designing and development of electrochemical biosensors enable molecule sensing and quantification of biochemical compositions with multitudinous benefits such as monitoring, detection, and feedback for medical and biotechnological applications. Integrating bioinspired materials and electrochemical techniques promote specific, rapid, sensitive, and inexpensive biosensing platforms for (e.g., point-of-care testing). The selection of biomaterials to decorate a biosensor surface is a critical issue as it strongly affects selectivity and sensitivity. In this context, smart biomaterials with the intrinsic self-assemble capability like bacterial surface (S-) layer proteins are of paramount importance. Indeed, by forming a crystalline two-dimensional protein lattice on many sensors surfaces and interfaces, the S-layer lattice constitutes an immobilization matrix for small biomolecules and lipid membranes and a patterning structure with unsurpassed spatial distribution for sensing elements and bioreceptors. This review aims to highlight on exploiting S-layer proteins in biosensor technology for various applications ranging from detection of metal ions over small organic compounds to cells. Furthermore, enzymes immobilized on the S-layer proteins allow specific detection of several vital biomolecules. The special features of the S-layer protein lattice as part of the sensor architecture enhances surface functionalization and thus may feature an innovative class of electrochemical biosensors.

## 1. Introduction

**Figure 1:**
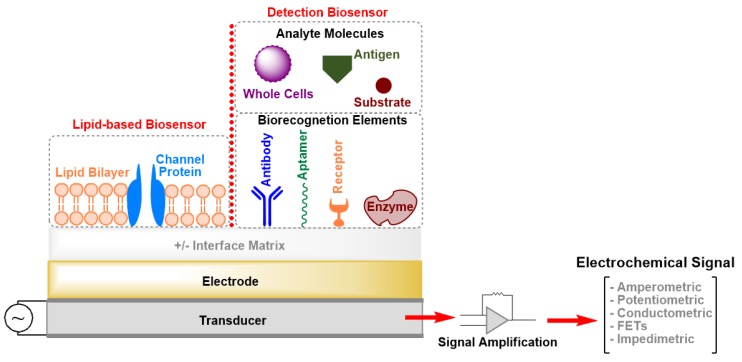
Schematic drawing (not drawn to scale) of the elements of an electrochemical biosensor. FETs: Field-Effect Transistors.

Biosensors are analytical devices that contain an integrated set of tools for the conversion of a biological response or an analyte concentration to specific (semi)quantitative information [1]. A typical biosensor is composed of three main parts (Figure 1): (i) Bioreceptors that specifically recognize an analyte and capture it; (ii) an interface matrix, which is a functional area where reactions occur; and (iii) a physico-chemical transducer element that converts the generated event into a (mostly electronic) quantifiable signal [1,2]. The detector unit can amplify the transducer signal before its transmission for processing and analysis by computer software. Several transduction mechanisms depend on the conversion of optical, acoustic, electrical, chemical, mechanical, or thermal properties, or a combination of different mechanisms, to detect biorecognition events [1,2]. All these techniques have led to the development of biosensors that are currently on the market and there is a great deal of interest in developing devices applicable as diagnostic tools for point-of-care (POC) testing. The main obstacles for the development of POC sensing devices with high sensitivity and selectivity are the complexity of the transduction principles and high production costs. Moreover, patients cannot use the POC devices by themselves, as skillful technicians are necessary to operate these instruments.

Electrochemical biosensors offer many advantages, including simplicity, low cost, short measurement time, requirement for only small volumes of analyte and reagents, and high selectivity and sensitivity. Moreover, easy operation is feasible that enables patients to handle electrochemical biosensors at home without the need for expensive facilities and personnel. Further, microelectronic circuits can not only be produced at low cost but can also easily connect with common electronic read-out and processing devices [2,3,4]. In contrast, electrochemical biosensors also possess some limitations. For example, although they make it possible to detect a target analyte in a complex biofluid sample, the pH and ionic strength can markedly interfere with the biochemical response signal. Further, the selectivity and sensitivity depend mainly on the architecture of the sensing layers. The design of the latter and the content of the biological recognition elements that are integrated within or connected to a transducer dominate the response to a specific analyte and subsequently control the biochemical event [2,5]. The biorecognition systems in biosensors should enable the formation of complementary structures with high affinity, such as antibody–antigen, enzyme–substrate, and receptor–ligand pairs. This is important because it is proportional to an analyte’s concentration [3]. Hence, there is a growing need to utilize nanotechnology at electrochemical biosensors in order to reduce the dimensions of the sensor elements and improve the signal-to-noise ratio. Moreover, exploiting (nano)biomaterials for functionalizing the sensor surface can significantly improve its sensitivity, especially to low-molecular-weight analytes. Several natural biomaterials provide support to improve the functionality of the sensing system. However, these materials must also satisfy particular criteria, such as being stable, flexible, biocompatible, and biodegradable. The biomaterials immobilized on the surface of a biosensor must not interact with the biological molecules to avoid any undesired physical–chemical interactions and, consequently, interference with the measured signal [6]. Promising biomolecules are surface (S-) layer proteins, which exhibit the natural feature to self-assemble into mono- or, if desired into guided double layers in solution and at various interfaces and surfaces [7,8].

**Figure 2:**
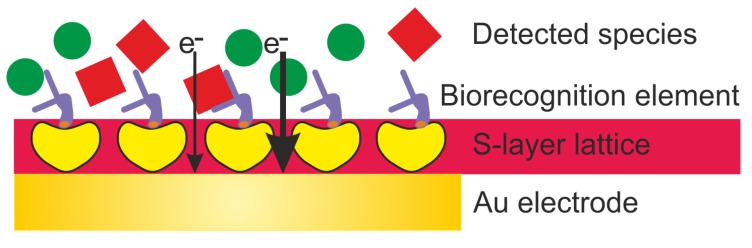
Schematic drawing (not drawn to scale) of an electrochemical biosensor with an S-layer lattice as intermediate layer for linking biorecognition elements to the Au (gold) electrode surfaces.

This review highlights the general approaches for the development of electrochemical biosensors and focuses on the exploitation of S-layer proteins to functionalize the sensing surface. The S-layer lattice constitutes a biocompatible intermediate layer for linking biological molecules to silicon oxide or metal surfaces and hence, decouples biological molecules from the electrode (Figure 2) [9,10]. This is of utmost importance as biological molecules may lose their structure and function on inorganic surfaces. The pores of the S-layer lattice allow unhindered electron transfer from and to the electrode surface and additionally provide an ion reservoir. 

## 2. Bacterial S-Layer Proteins

### 2.1. General Features

**Figure 3:**
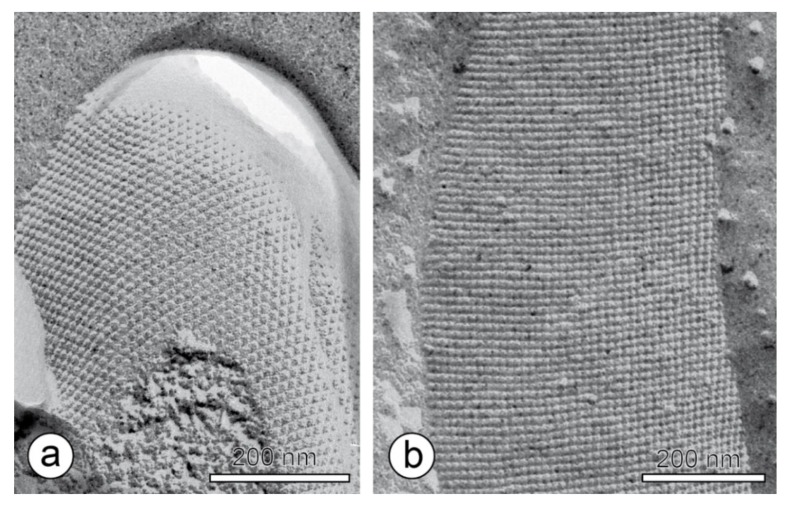
Transmission electron microscopy image of a freeze-etched and metal shadowed preparation of (**a**) an archaeal cell (from *Methanocorpusuculum sinense*), and (**b**) a bacterial cell (from *Desulfotomaculum nigrificans*). Bars, 200 nm. With permission from Sleytr et al. 2014 [7] (CC BY-NC-ND 3.0).

An S-layer is per definition a “two-dimensional array of proteinaceous subunits forming the surface layer on prokaryotic cells” (Figure 3) [7]. The S-layer covers the entire cell surface as a closed layer [11,12,13,14], shapes the outermost structure of many bacteria, and are a nearly ubiquitous characteristics of archaea [7,15,16,17,18]. A frequent post-translational modification in S-layer proteins is protein glycosylation of certain amino acid residues, which is a remarkable characteristic of many archaeal and some bacterial S-layer proteins [16,19,20,21,22]. Table 1 summarizes the most important features with respect to its application as intermediate layer, ion reservoir and immobilization matrix in biosensor development.

S-layer proteins have so far suggested to mediate a broad range of specific biological functions, including protection against (e.g., bdellovibrios, bacteriophages, and phagocytosis) promoters for cell adhesion (e.g., to host enzymes and cells, immune-modulators, surface recognition, molecular sieve, molecule and ion traps, antifouling coatings, and virulence factors in pathogenic organisms) [7,23,31,32]. Moreover, the S-layer lattice is involved in the determination of cell shape and can aid in the cell division process in archaea possessing S-layers as the exclusive envelope component external to the cytoplasmic membrane [32,33,34,35]. 

The most significant characteristics of S-layer proteins is the intrinsic ability of natural and recombinant protein subunits to self-assemble on surfaces or interfaces into crystalline arrays. These materials comprise glass, silicon oxide and nitride, mica, noble metals like gold, titan, platinum, but also of stainless steel or many polymers as polystyrene, polyester, and cellulose, and technically relevant materials like highly oriented pyrolytic graphite (HOPG) or indium tin oxide (ITO) [28,29]. Initiation of crystal growth at interfaces (e.g., solid supports, air–water interface or lipid membranes) occurs simultaneously at many randomly distributed nucleation points, and proceeds in plane until the crystalline domains meet. This finally results in the formation of a closed, coherent mosaic of individual, several micrometer large S-layer protein patches within 45 to 90 minutes [36,37,38,39]. The growth of extended S-layers patches is favored at low monomer concentrations due to the corresponding low number of nucleation sites. The individual patches are monocrystalline and separated by grain boundaries. However, these S-layer protein patches form a coherent lattice thereby coating areas in the cm^2^-range.

In general, S-layer protein lattices constitute a unique immobilization matrix for presenting diversified biomolecules in nanometer distances [7,40,41]. Moreover, the S-layer protein lattice acts as a versatile scaffolding for the formation of supported lipid membranes. The latter provide the essential ambience for the insertion of membrane-active peptides and the reconstitution membrane proteins [9,42,43,44].

### 2.2. Antifouling Properties

An important biological function of the S-layer lattice is to prevent fouling of macromolecules on the cell surface. Electron micrographs showed no adsorbed molecules on the S-layer lattice even for cells harvested from complex environments or growth media containing a great variety of macromolecules [11]. In addition, S-layers from thermophilic bacteria did not adsorb charged macromolecules on their surface or inside the pores because this would hinder the transport of nutrients and metabolites [45,46,47]. Based on these results, S-layers proteins can be considered as structures with excellent “antifouling” properties.

The antifouling characteristics of the S-layer protein lattice has not only investigated in vitro with S-layer ultrafiltration membranes (SUMs) [48,49,50], but also with coatings in electrochemical microfluidic biochips [28]. Cyclic voltammetry (CV) measurements using ferricyanide/ferrocyanide (FCN; [Fe(CN)_6_]^3−/4−^) as the redox system allowed the determination of the peak current of gold electrodes with and without an S-layer lattice cover in the presence of human serum albumin (HSA; 30 mg/mL). Interestingly, HSA adsorption led to a much higher clogging of the plain gold electrode, as the CV measurements resulted in a more than 96% reduced peak current for the plain electrode (0.3 µA), as compared to the electrode with the recrystallized S-layer protein (12,8 µA) [28]. Moreover, surface plasmon resonance (SPR) analysis showed that the adsorption of HSA (60 mg/mL) and human blood components is much higher for plain gold surfaces (3.74 and 4.44 ng/mm^−2^ for HSA and human blood, respectively) than for S-layer protein covered gold surfaces (0.18 and 0.46 ng/mm^−2^ for HSA and human blood, respectively) [28]. Hence, these two in vitro studies demonstrate imposingly the antifouling characteristics of S-layer protein lattices on metal electrodes. 

The antifouling property of the S-layer protein lattice was also compared to other favored immobilization supports, which are polyethylene glycol (PEG) and bovine serum albumin (BSA). As determined by SPR, the S-layer lattice showed again the lowest amount of adsorbed HSA (0.18 ng/mm^−2^) followed by PEG (0.76 ng/mm^−2^) and BSA (1.92 ng/mm^−2^) [28].

### 2.3. Electrochemical Properties

**Figure 4:**
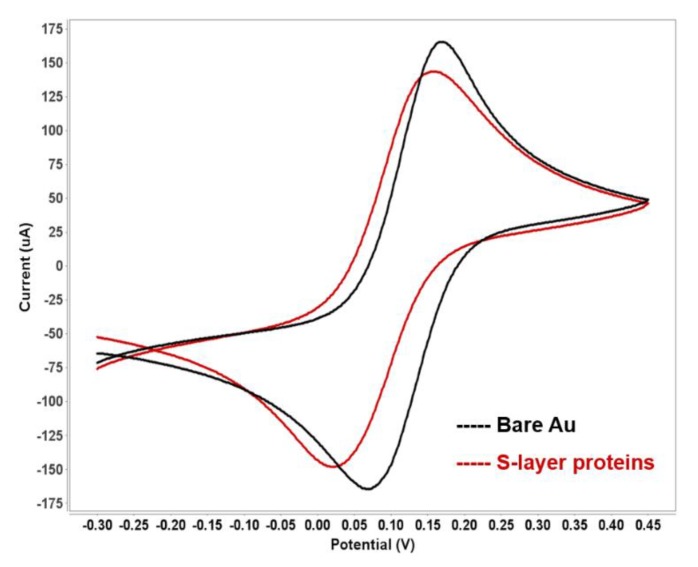
Cyclic voltammogram of the bare and the S-layer protein-coated gold electrode in 10 mM [Fe(CN)_6_]^3−/4−^ containing 100 mM KCl at a scan rate of 50 mV/s.

As previously mentioned, the S-layer lattice is a porous structure with a porosity up to 70% [7,24]. The latter feature additionally provides an ion reservoir. Figure 4 shows the cyclic voltammogram of an S-layer protein recrystallized on a gold electrode in the presence of the FCN redox system and 100 mM KCl. The bare electrode surface has the highest peak current, which indicates a small resistance in the electron transfer. Subsequently, recrystallization of the S-layer protein onto the gold sensor leads to a slightly reduced peak current, which attributes to a decrease in the electron transfer resistance. Thus, the S-layer lattice reduces the electrode area to some extent, but the porous intermediate layer allows for efficient redox-mediator diffusion and unhindered electron transfer from and to the electrode surface, thus the sensitive detection of subsequent biorecognition events. The resistance and capacitance of the S-layer lattice itself is negligible. Hence, further binding events of analytes or (cancer) cells at different quantities results in measurable changes in the electrochemical properties of the sensing layer.

In general, proteins are biopolymers comprised of amino acids. That is why it is not surprising that the electrical properties of the surface onto which the S-layer protein is self-assembled has a pronounced effect on the lattice formation. The electrochemical behavior of the S-layer SbpA isolated from *Lysinibacillus sphaericus* CCM 2177 on gold electrodes was studied, beside spectroscopical and microscopical techniques, by in-situ electrochemical quartz crystal microbalance [51,52,53]. 

The self-assembly and bond formation of S-layer proteins on a gold surface can be affected and controlled by direct electrochemical manipulation. The S-layer proteins self-assemble to a crystalline lattice in the positively-charged electrochemical double layer region where solvated anions form the electrochemical double layer. In this region, the carboxyl groups of the S-layer protein can interact with the positively-charged gold atoms on the electrode surface. In contrast, there is no lattice formation in the negatively-charged electrochemical double layer region [51]. In another study, the S-layer lattice of *Deinococcus radiodurans* shows ion-gating properties as demonstrated by EIS [52]. Ion transport appears to be mainly due to an electrical gradient inside the pores, presuming to originate from the negative charges, which are present on this S-layer lattice. A detailed study on the gating characteristics of this nanoporous structure toward various ionic species suggested, that the immobilized S-layers experience a strong interaction with cations, particularly Ca^2+^-ions. The latter interlink laterally with the S-layer proteins lattice in the positively-charged electrochemical double layer region, thus facilitate the formation of the crystalline S-layer protein lattice [53,54].

An aluminum—S-layer protein (from *Lactobacillus brevis* ATCC 8287)—ITO/polyethylene terephthalate device structure allowed the examination of the nonvolatile memory characteristics of this S-layer protein by CV [55]. The S-layer protein is very interesting for this approach, beside other intrinsic features, because it is redox inactive. It turned out that the nonvolatile resistive switching characteristics of the S-layer protein can be utilized for active memory storage in a powered-off state of electronic chips. Thus, this flexible memory device could find a way in wearable storage applications like smart bands and sports equipment sensors [55].

## 3. Basic Principles of Electrochemical Biosensors Used in Combination with S-Layer Proteins

**Figure 5:**
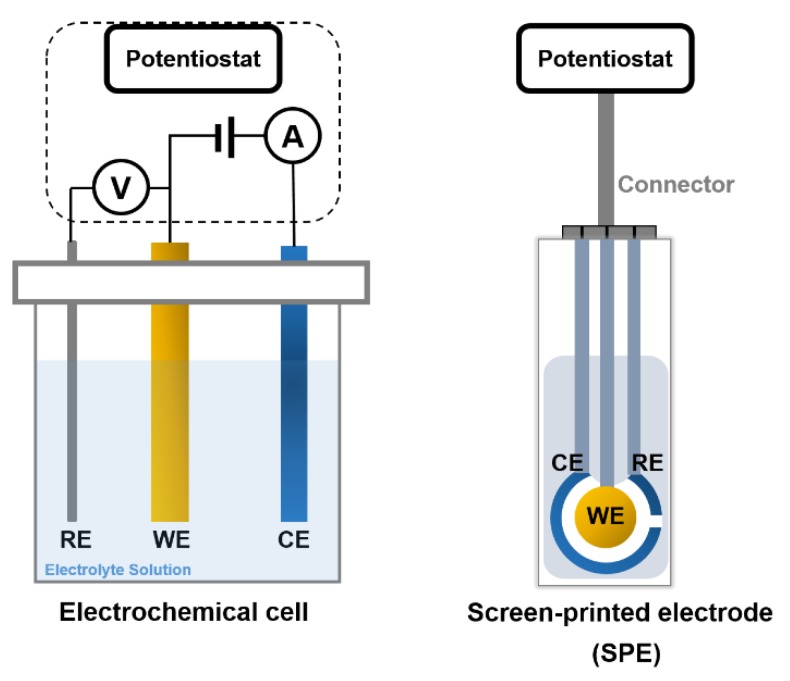
The two types of electrochemical biosensors with three electrodes: reference (RE), working (WE), and counter (CE) connected to a potentiostat.

Electrochemical measurements depend on the spontaneous interaction between electrical energy and a chemical reaction involving an oxidation–reduction reaction to generate an electrical current or vice versa. The chemical events that occur between immobilized biomaterials and the analytes result in the production/consumption of ions or electrons, which has an effect on the electrical current, the electrical potential, or any other electrical property of the solution. These reactions take place at the interface of a metal or semiconductor electrode and an electrolyte [56,57,58]. Thus, detection is feasible if the reactions occur in close contact with the electrode surface. Hence, the electrodes significantly influence the performance of the electrochemical biosensor. One has to consider several factors when choosing a proper electrode, including its material, dimension, and possibility to carry out surface modifications. Most electrochemical cells are composed of three electrodes (Figure 5):

A reference electrode (RE): This electrode is usually made of Ag/AgCl and stands at a distance from the place where the reaction occurs to provide a potential that is proportional to the known and stable solution. The RE allows normalizing of the measurements.

A counter (auxiliary) electrode (CE): This electrode is the source of the current, which is subsequently applied to the working electrode.

A working electrode (WE) (the sensing or redox electrode): This electrode acts as the transduction element in the biochemical reaction. CE and WE should be conductive and chemically stable. Thus, depending on the analyte and the nature of the reaction, the mainly used electrode materials are gold, silver, platinum, silicon, carbon, and graphene. An alternative to electrochemical cells is to screen-print the three electrodes on an insulating substrate. These so-called screen-printed electrodes have several advantages, including simplicity, ease of mass production, low-cost construction, and low analyte/reagent consumption [2,59,60]. 

Classification of electrochemical biosensors occurs according to their characteristics, such as biorecognition element, the biological mechanisms, the electrochemical detection technique, or combinations of these approaches. In the following section, we briefly discuss classes of electrochemical biosensors based on the mode of signal transduction. Modification on some types of these electrochemical biosensors occurred either by the self-assembly of isolated S-layer protein subunits on the sensor surface or by using a SUM.

### 3.1. Amperometric Biosensors

Amperometry means to measure the current that results from the electrochemical oxidation or reduction of an electroactive species. Applying a constant potential at the WE or on an array of electrodes with respect to the RE induces the generated current, which is associated with the redox process. In amperometric devices, the term “amperometry” refers to the technique characterized by monitoring the current at a constant potential. In turn, the term “voltammetry” refers to a technique monitoring the current during variations in the potential over a certain potential range. In both techniques, the measurable current correlates directly with the production/consumption rate of the electroactive species or to the bulk analyte’s concentration [1,2,6,61]. In this amperometric approach, also SUMs and S-layer proteins recrystallized directly on the gold electrode constitute key components for the immobilization of enzymes (see Table 2) [28,62,63]. 

### 3.2. Potentiometric Biosensors

Potentiometric devices monitor the accumulation of a charge potential either at the WE compared to the RE or between two REs separated by a semipermeable membrane. Monitoring the potential occurs in an electrochemical cell under the condition that no significant current flows between the two electrodes. The transducer is usually an ion-selective electrode (ISE) composed of an electrochemical sensor with a recognition element based on a thin film or a selective membrane. The conversion of the biorecognition event into a potential signal by the ISE gives rise to a measurable signal [64].

### 3.3. Conductometric Biosensors

Conductometry is based on the relationship between a biorecognition reaction and conductance. Changes in the concentration of ionic species or of ionic strength in the sample result in an alteration in the electrical conductivity of the solution or in the current flow. Hence, conductometric devices measure the capability of an analyte or a medium, such as an electrolyte solution or nanowires, respectively, to conduct an electrical current between two metal electrodes (e.g., Ag or Pt). An ohmmeter measures the changes in conductance between these electrodes. Some studies consider conductometric biosensors to be a subset of impedimetric biosensors. However, conductometric and impedimetric devices constitute convenient sensors to investigate enzymatic reactions that generate changes in the concentration of charged species in a solution [3,65,66]. Both techniques use nowadays interdigitated microelectrodes where the enzymatic reaction results in changes in the ionic strength and conductivity of a solution between two electrodes. 

### 3.4. Field-Effect Transistors (FETs)

FETs are devices composed of three electrodes: source, gate, and drain. These types of transistor use an electric field to control the conductivity of a channel where a depletion region of charge carriers lies between the source and drain electrodes in a semiconducting material. Variations in the electric field potential relative to the source and drain electrodes at the gate electrode contribute to controlling the conductivity. The configuration and doping of the semiconducting material govern the charge carrier’s behavior in the conduction channel, i.e., at the gate electrode. The presence of an appropriate positive or negative potential may either attract or repel charge carriers, such as electrons, in the conduction channel. This may result in the filling or emptying of the depletion region of charge carriers and, subsequently, form or deform the effective electrical dimensions of the conduction channel. Hence, the conductance between the source and drain electrodes can be controlled. There are several types of FET devices for the measurement of ion concentrations; however, the most popular are ion-sensitive field-effect transistors (ISFETs) and enzyme field-effect transistors (EnFETs) [1,2,67]. 

### 3.5. Impedimetric Biosensors

Impedance techniques are efficient tools for measuring alterations in electrical properties that result from biorecognition reactions at a modified electrode’s surface. Electrochemical impedance spectroscopy (EIS) is one of the most useful tools for constructing biosensors that can monitor the current response to an applied potential. Complex impedance is the sum of the real and imaginary impedance components, resistance and reactance, respectively, of a system as a function of the angular frequency (ω). Calculation of the impedance occurs by altering the excitation frequency (*f*) of the applied potential over a range of frequencies. EIS can be used to investigate the properties of a material or specific processes that can affect the capacitivity or conductivity/resistivity of an electrochemical system [2,4,68]. EIS technique is widely used in electrochemical biosensors based on S-layer proteins for several applications to assess membrane-active peptide insertion and membrane protein reconstitution into artificial lipid membranes and specific cell detection [69,70,71,72,73].

## 4. Sensor Surface Modifications

**Figure 6:**
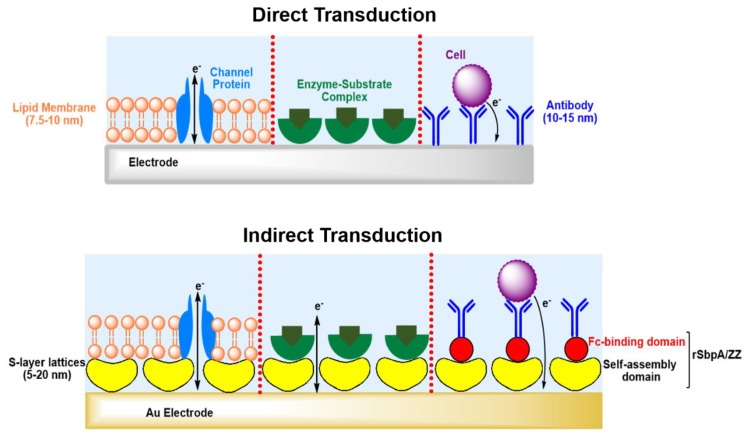
Direct and indirect transduction (not drawn to scale). In direct approach, the electron transfers are close to the surface, whereas in the indirect one, electron shuttles between the reaction site and the sensor surface. In the proposed model, the S-layer protein lattice constitutes an intermediate matrix. In the lipid-based biosensor (left), electrons transfer from the outer membrane to the inner membrane and vice versa via a channel protein. In the detection biosensor (middle and right), electrons transfer between the enzyme–substrate complex and cell/antibody and electrode surface, respectively. The S-layer lattice provides an immobilization matrix and ion reservoir. The pores of the S-layer lattice ensure no impact on the electron transfer. Fc: fragment crystallizable; rSbpA/ZZ: recombinant S-layer protein from *Lysinibacillus sphaericus* CCM 2177 with fused Fc-binding Z-domain (synthetic analog of immunoglobulin G (IgG-binding B—domain) of protein A of *Staphylococcus aureus*).

The construction of an ideal biosensor involves two areas: bioactive and inert. The bioactive area is the region where the biochemical reactions and detection events take place. The inert area is the one, which surrounds the bioactive one. Both areas must be composed of carefully selected elements in order to avoid interference of reactions and non-specific adsorption. The bioactive surface needs a smart design to improve the sensing sensitivity and to minimize sample consumption. However, functionalization of an active surface requires conquering several challenges. The molecules that are adsorbed/attached onto the surface must be in a suitable pattern and at an appropriate density to avoid an insufficient number of molecules or crowding on the sensing area, which may influence analyte capture and the sensor’s functionality (Figure 6). Further, the developed biosensor must be stable and not be affected by environmental conditions such as time frame and storage.

Materials to prepare the sensing substrate are manifold. However, these materials must fulfil the special requirements of electrochemical measurements, such as electrical conductivity. Most suitable substrates are carbon, graphene, carbon nanotubes, gold, and platinum, depending on the measurement technique and nature of the analyte itself. For example, carbon nanotubes offer many advantages, including good conductivity, high chemical stability, a high surface area, and high mechanical strength [74]. Further, metallic surfaces, such as a gold surface can be modified with self-assembled monolayers (SAMs) of thiols in order to allow molecules to be adsorbed in a highly ordered and well-organized pattern while maintaining electron diffusion. Furthermore, during fabrication of an electrochemical biosensor, it is usually favorable to coat the WE with an extra layer as a spacer or mediator to enhance the immobilization of biomolecules in a well-oriented manner. The intermediate layer can provide another advantage, which is to minimize non-specific binding. Polymers (e.g., chitosan, agarose, hydrogels, etc.) and proteins (e.g., S-layer proteins) are convenient to functionalize sensor surfaces and exhibited a stable matrix for a lipid-based biosensor suitable for studying and utilizing membrane protein as sensing elements [9]. In contrast, the intermediate layer influences the sensitivity of the sensor because an increase in the distance to the surface corresponds to a decrease in the signal (Figure 3). In electrochemical biosensors, the reaction needs to occur close as possible to the electrode surface because the products can diffuse in many directions away from the surface, which can contribute to a lower signal. Thus, a careful choice of the spacer or mediator is necessary to ensure that these molecules act as a shuttle transferring electrons between reaction site and electrode surface [2,75,76].

The fabrication of lipid membrane-based biosensors used with electrochemical techniques depends mainly on the composition of the lipids and proteins. Biological membranes are the most important electrified interfaces in living systems. Due to the hydrophilic head and hydrophobic tail, lipid molecules are amphiphilic and, hence, can be self-assembled to form a bilayer that consists of two monolayers. In this configuration, the non-polar tails point toward the hydrophobic interior of the bilayer and polar head is oriented toward the external aqueous surrounding. The generated lipid matrix can further host proteins that act as receptors, pores or channels. Natural and synthetic membranes are selective to permeable ions that cross between the inside and outside of membranes. Further, high electrochemical potentials can be maintained across the interior and exterior monolayers [72,73,77,78]. The chemical nature of the membrane components and the reactions that occur at the interface of the monolayer or within the bilayer govern the permeability and structural properties of the membrane. Many membrane proteins in lipid membranes are electrogenic and translocate a net charge across the membrane. Thus, monitoring the functions of membrane proteins occurs directly by measuring the flow of current along an external electrical circuit when these protein molecules are in an activated state. Further, monitoring of deactivation of proteins occurs also by tracking the drop in current after addition of a chemical agent, such as a drug (agonist or antagonist) [77]. A well-known model that shows how the electrical and permeability characteristics of a lipid membrane and its protein content are affected is the partition of anesthetics into a lipid membrane. The response of a biomembrane to the presence of anesthetics or toxins is usually rapid, reversible in a concentration-dependent manner, and may be voltage-dependent. The first step in building a lipid membrane-based biosensor is the choice of the lipid composition. A natural cell membrane is negatively charged and, hence, exploiting negatively-charged lipids is a challenging approach to create artificial but biomimetic lipid membranes. The choice of lipid is critical as it may further influence the protein reconstitution and membrane activity [79]. The generation of a synthetic membrane on a negatively-charged surface, such as mica or silicon can result in repulsive electrostatic interactions that disturb lipid bilayer formation, [80,81]. This is in particular the case if the pH and ion strength of the surrounding solution is not adjusted appropriate [80]. However, the addition of positively-charged molecules, such as positively-charged lipids and peptides/proteins may foster the integrity to the membrane. Moreover, alterations in the pH affect the degree of ionization, resulting in changes in lipid pKa values and consequently the charge of the lipid molecule [81,82]. Insertion of membrane-active peptides is in general not difficult as these peptides are soluble (e.g., in alcoholic solutions). The insertion of membrane proteins is a big challenge, as with few exceptions membrane proteins have to be solubilized by means of detergents [83]. The reconstitution of membrane proteins in a synthetic membrane, however, can nowadays also be performed either by cell-based or cell-free protein expression systems. Each system offers advantages and disadvantages as extensively discussed in a number of publications [84,85,86]. The formation of a lipid membrane-based biosensor can occur directly on a metal electrode or indirectly by a matrix layer. The latter is favorable as it can minimize direct contact between the reconstituted membrane protein and the substrate, which can further minimize protein denaturation. Additionally, it represents a membrane model that is robust, highly hydrated, and has long-term stability. 

Another application that exploits an electrochemical technique is detection biosensors. These devices inherently possess biorecognition elements that specifically recognize a target analyte. The biorecognition molecules can be immobilized directly onto the WE by physical adsorption or onto a modified matrix based on chemical interactions. This should ensure correct orientation and to minimize random adsorption, which can further improve the capture efficiency. Antibodies, nanobodies, aptamers, affimers, and receptors are examples of used biorecognition elements with high affinity and specificity for particular antigens and cells [87]. Quantitatively, the formation of a complex between the biorecognition molecule and its target results in a measurable signal. Moreover, biosensing devices expedite the exploitation of enzymes as electrocatalysts. Oxidation or reduction reactions with the substrate generate products, which give rise to a detectable signal. Similar to detection sensors with biorecognition molecules, enzymatic biosensors can be constructed either by direct enzyme adsorption onto the sensor surface or on a mediator/intermediate matrix. The most common enzymatic biosensors on the market are the ones composed of glucose oxidase for the detection of blood glucose levels in diabetic patients [33,88,89,90].

## 5. Application of S-Layer Proteins in Biosensors

### 5.1. S-Layer Protein on Gold Surfaces

The utilization of S-layer proteins as the sensing element for detecting metal ions using EIS is a straightforward approach. The immobilization of the S-layer protein from *L. sphaericus* JG-A12 on a gold surface occurred by covalent binding of intrinsic cysteine residues of the S-layer protein to a SAM presenting terminal maleimide groups (Table 2). Another possibility is to link the biotinylated S-layer protein via neutravidin to a mixed SAM that presents biotin molecules as well [91]. The single intrinsic S-layer proteins immobilized on the electrode surface acted as specific binding sites for aqueous uranyl ions (UO_2_^2+^). In this approach, the S-layer proteins have not assembled to a crystalline lattice. Both, phosphate and carboxyl groups of the S-layer protein are involved in the recognition to UO_2_^2+^. The limit of detection for UO_2_^2+^ ions was determined to 10^−12^ M. Moreover, minor interference from Ni^2+^, Cs^+^, Cd^2+^, and Co^2+^ ions are detectable. [91]. A straightforward approach might be to collect S-layer carrying organisms from metal polluted environments in order to use their isolated S-layer proteins as sensing elements for the detection of heavy metal ions or radioactive ions. This is in particular a field of application for monitoring environmental pollution. 

### 5.2. S-Layer Protein and Enzymes 

**Figure 7:**
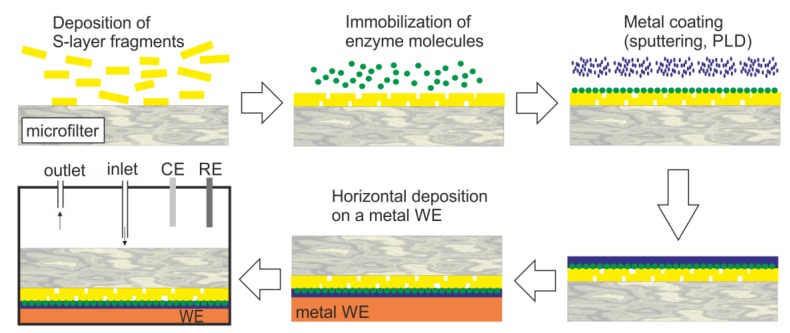
Generalized scheme of the construction of an S-layer ultrafiltration membrane (SUM)-based biosensor (not drawn to scale). S-layer carrying fragments or self-assembly products are deposited on a commercially available microfilter in a pressure-dependent process. The enzyme is deposited on the S-layer surface and chemically linked to the S-layer protein. The immobilized enzyme is contacted by a thin metal layer (PLD: pulse-laser-deposition). Finally, this composite structure is deposited with the metal layer side on the working electrode (WE) and mounted in a flow cell (RE: reference electrode, CE: counter electrode). The analyte is pumped in the flow cell. After passage across the modified SUM, the analyte reacts with the enzyme, which is detected by amperometry.

The intrinsic permeability characteristics of self-assembled S-layer protein lattices of diverse Bacillaceae is useful to produce SUMs, which serve as isoporous molecular sieves with a pore dimension of 4 to 5 nm [45,46,48,49,50]. SUMs are not only proper structures for filtration applications where a sharp cut-off is needed, but also suitable as immobilization matrix for functional molecules (Figure 7). Beside enzymes (glucose oxidase (GOx), β-glucosidase, glucuronidase, cholesterol esterase and oxidase, mutarotase, invertase, maltase, xanthine oxidase, alcohol oxidase, naringinase, peroxidase, laccase, etc.), also ligands (protein A, streptavidin) or mono- and polyclonal antibodies have been immobilized as densely-packed sensing layers on SUMs [7,47,48,63,94,95].

The first amperometric sensor based on an S-layer protein lattice, developed 27 years ago, was comprised of an SUM with covalently bound GOx [62]. Table 2 shows a summary of the performance of SUM-based biosensors. The immobilization of the GOx on the SUM caused an activity reduction to approximately 40% [62]. Hence, improvements were necessary. In a first attempt, the transducer was modified to an oxygen optode with optical read-out system [96]. The recrystallization of an S-layer protein improved the fiber optic probe as the GOx molecules could be bound in a very close proximity and its tightest packing. The next improvement was to replace the argon sputtering method for metallization of the composite S-layer protein/GOx layer by the pulse-laser-deposition technique (Figure 7) [97]. Indeed, this technique achieved to retain the enzyme activity to up to 80%, which represents a two-fold GOx activity with reference to the first biosensor utilizing the S-layer protein lattice as binding matrix for the GOx molecules (Table 2) [62].

The continuous, stable, and accurate determination of glucose in biological fluids is a challenging approach particularly in blood because many constituent parts interfere the measurement and monitoring. S-layer protein lattices provide highly hydrated, nm-thick, biological antifouling materials [98]. Moreover, SbpA has the ability to form monomolecular lattice structures at various microchip surfaces (e.g., glass, silicone oxide, polydimethylsiloxane, platinum, and gold) within one hour. To utilize these features, the idea came up to develop a microfluidic device consisting of four individually addressable amperometric biosensor arrays, each coated by an S-layer lattice [28]. In this arrangement, the S-layer lattice has multiple tasks, which are to offer a reduced unspecific binding of blood components, an anchoring structure for the enzyme in a way that the catalytic sites of GOx are accessible, and to act as an efficient molecular sieve. The flow cell configuration made it possible to measure simultaneously the concentration of the glucose in blood, perform auto-calibration routines, detect mediator-interferences, and subtract the background. The lab-on-a-chip device precisely measured the glucose concentration in blood (Table 2). The latter is in particular important during hemodialysis because it allows individual balancing of the glucose level. The highly porous S-layer protein coating eliminated unspecific adsorption events in the presence of human serum albumin, human plasma and freshly drawn blood samples. Most important, the undisturbed diffusion of the mediator to the electrode surface enabled bioelectrochemical measurements of glucose in the concentration range of 500 μM to 50 mM [28]. 

To generate a biosensor for sucrose (Table 2), immobilization of the three enzymes GOx, mutarotase, and invertase on S-layer protein self-assemblies from *Clostridium thermohydrosulfuricum* L111-69 occurred using spacer molecules comprising aspartic acid [94]. The S-layer protein self-assemblies carrying the different enzymes were mixed and deposited on the microfilter. This resulted in a final structure shown in Figure 7c, but with three immobilized enzymes. Hence, this is a further example demonstrating that sandwich structures comprising recrystallized S-layer protein/enzyme/metal layer constitute seminal biosensor systems with high efficiency. 

In order to measure cholesterol in biological fluids, biosensors often take advantage of the enzyme cholesterol oxidase (ChOx) [63,99]. A coating method that has proven itself as very useful and straightforward is based on the production of monomolecular films at the air–water interface. It was possible to generate films out of ChOx and mixtures of ChOx with S-layer proteins at the water–air interface [100]. The mixed films showed the advantage of being stable over a longer period compared to the pure ChOx films. The Langmuir–Blodgett transfer of mixed ChOx/S-layer protein films on screen-printed carbon electrodes enabled to study their possible application in biosensor devices (Table 2) [95]. Atomic force microscopy demonstrated the successful transfer of films, which caused a reduction of the roughness of the electrode surface as well. CV measurements revealed increased oxidation peak intensity and reduced oxidation potential for the mixed films compared with the pure enzyme films. Moreover, the presence of S-layer proteins reduced the potential where oxidation occurs, making the sensor more selective, since a higher potential will oxidize non-wanted species, which can interfere with cholesterol detection. Hence, screen-printed carbon electrodes combined with a monomolecular ChOx/S-layer protein film constitute a promising biosensor for measuring cholesterol [95].

### 5.3. Biosensor for Sensing Cells

The lack of effective diagnostic tools contributes directly to the high mortality rate of cancer. Early detection of cancer can significantly contribute to an effective treatment and increase the survival rate of patients. The currently used diagnostic techniques include polymerase chain reaction, flow cytometry, immunohistochemistry, immunofluorescence, and mass spectrometry. These assays are usually costly, time-consuming, labor-intensive, require several steps and multiple reagents, and suffer from some limitations to their selectivity and sensitivity. In contrast, electrochemical biosensors have attracted attention as a promising alternative to the conventional methods. An additional advantage that electrochemical technology provides is the ability to detect a whole cell or an intact cell by identifying the expression of a surface marker on the cell membrane, which excludes the requirement for pre-processing steps. Moreover, besides their ability to detect cancer cells, cell-based biosensors provide an efficient and simple approach for the investigation of cell viability, cell proliferation, and the effect of chemotherapeutic drugs on cancer cell viability [101,102]. Several successful studies describe the capture/recognition of cancer cells at the electrode surface using various electrochemical methods, including CV [103], square wave voltammetry (SWV) [104], differential pulse voltammetry (DPV) [105], and EIS [106].

The careful design of the interface architecture of an electrochemical biosensor is very important to ensure high performance, sensitivity, and selectivity. The main factor that restricts the biosensor efficiency is the orientation and density of the affinity biomolecules (recognition elements (e.g., antibody, nanobody, aptamer) and antigens (e.g., cell)) on the sensing area [107,108]. For example, immobilization of antibodies onto WEs is possible by approaches that involve physical adsorption and chemical interactions. Despite the extensive use of these methods, they still suffer from serious drawbacks. Physical adsorption can result in the immobilization of antibodies in a random orientation, while covalent coupling based on chemicals (e.g., glutaraldehyde, carbodiimide) or a self-assembled monolayer (e.g., nanoparticles, protein A, protein G) may alter the biological activity of the antigen-binding sites of the antibody [8,109,110].

To overcome these limitations, the recombinant S-layer fusion protein rSbpA/ZZ has been exploited to develop a detection biosensor for circulating tumor cells (CTCs) that overexpress CD133. CD133 is a five-transmembrane, single-chain glycoprotein expressed in various human epithelial carcinomas, including lung, colon, liver, and gastric carcinoma [111]. Functionalization of the biosensing area of the fabricated sensor occurred by immobilization of rSbpA/ZZ and anti-CD133 antibodies. rSbpA/ZZ has two copies of the 58-amino-acid-long Fc-binding Z-domain, a synthetic analog of the immunoglobulin G (IgG)-binding B-domain of protein A of *Staphylococcus aureus* [112,113]. The protein A binding site of rSbpA/ZZ helps to immobilize the antibodies (IgG) in the correct orientation [113]. Thus, rSbpA/ZZ, when used to generate a uniform matrix on a gold electrode, enables the immobilization of anti-CD133 antibodies in the correct orientation to enhance the recognition and capture of the surface marker CD133, which is expressed on membranes of hepatic cancer stem cells [72]. The CV technique allowed evaluation of the functionality of the developed sensor. Voltammetric measurements converted the interactions between the anti-CD133 antibody and the hepatic cells into an electronic signal. The peak current decreased consistently in response to an increasing number of captured cells on the electrode surface (Table 2). The importance of the development of biosensors for the detection of CTCs derives from their roles in cancer diagnosis, as they can provide an evaluation of a treatment strategy, its effectiveness, and the possibility of cancer recurrence after treatment. The main obstacle for the capture of CTCs is their limited number in the blood (1–100 cells/mL blood) [114].

In a recent study, modification of the S-layer protein SbpA occurred by chemical binding of folic acid. This SbpA-folate construct self-assembled to form a monomolecular S-layer lattice on a gold surface [73]. The S-layer protein lattice offered an even distribution of the folate residues on the surface of acoustic and electrochemical biosensors. This arrangement ensured the specific capture of human breast adenocarcinoma cells (MCF-7), which expose folate receptors on their cell surface. Indeed, clear differentiation between human liver hepatocellular carcinoma (HepG2) cells, which do not express folate receptors and MCF-7 cells, was feasible (Table 2). The thinness and antifouling characteristics of the SbpA lattice caused an increased efficiency for cell capturing and no need to block the surface to prevent unspecific binding, respectively. Beyond, there is no necessity for immobilizing antibodies because the folate residues constitute an alternative to antibodies for capturing target cells. Over all, evaluation of the developed biosensors by different techniques provides more information about the efficiency of the system. QCM-D measurements tracked the formation of SbpA-folate modified sensor and capturing of cancer cells efficiently in real-time and under controlled conditions. Although the QCM-D technique shows a limited detection range, it allows tracking the cell viability [73]. Hence, it is worth to perform further QCM-D studies to elucidate the cellular response to chemotherapeutic agents. In addition, electrochemical recordings substantiate the selectivity and specificity of cell binding by this engineered biosensor. SWV measurements demonstrated a direct relationship between the peak current and the number of cells captured on the sensor surface. The peak current increased to a more negative value as the cell density increased, indicating a rising coverage of the electrode’s surface by the captured cells [73]. Interestingly, the same density of HepG2 cells, which lack the folate receptor, did not show a significant change in the peak current. Thus, modifying biomaterials that enable the presentation of ligand molecules in an optimal density and orientation can result in efficient biosensors with high selectivity.

Continuing research to identify efficient recognition elements and careful design of surface architectures may help us to make further progress in the fabrication of biosensors. This, in turn, may provide us with functional platforms to detect cancer and assist with early diagnosis. Moreover, this biosensor offers a straightforward, cost-efficient and disposable check-up for the early detection of cancer. 

### 5.4. S-Layer Protein and Functionalized Lipid Membrane

Archaea, which constitute one of the three evolutionary domains that make up all life on our planet are small single-celled organisms. They thrive in extremely harsh environments on Earth, such as sulfuric hot springs or deep-ocean hydrothermal vents that reach 386 K under colossal pressure [79,115,116,117]. The cell envelope structure of some archaeal species (e.g., *Sulfolobus* spp.) constitute simply of a cytoplasm membrane comprising of ether lipids and a membrane-anchored S-layer lattice [17,79]. The latter may provide mechanical and osmotic cell stabilization [118,119]. In a biomimetic approach, the cell envelope structure of archaea constitutes the construction manual for S-layer-supported lipid membranes (SsLMs) [17,32,79]. Due to the unique interaction of archaeal S-layer proteins with the cytoplasm membrane, no suitable methods for disintegration of archaeal S-layer protein lattices and the reassembly into monomolecular arrays on lipid films are available. Thus, the building blocks for the generation of SsLMs are isolated or chemically-synthesized phospholipids and isolated or genetically-produced S-layer proteins from Gram-positive bacteria (Figure 6, indirect transduction, outer left part) [9,42,43,44,120,121]. 

With respect to biosensor development, a stable lipid membrane is a prerequisite to make use of membrane-active peptides and membrane proteins as sensing elements. Indeed, many studies have proven that the attached S-layer lattice increases the mechanical stability of the lipid membrane significantly without impairing the fluidity of the membrane [9,42,43]. Membrane-active peptides as well as membrane proteins need the hydrophobic matrix to be able to adopt their native structure and hence, their functionality. The porous S-layer lattice functions as (1) stabilizing and anchoring scaffold for the lipid membrane; (2) biocompatible layer linking biological molecules with silicon oxide or metals; (3) intermediate protein layer, which decouples the lipid membrane from the solid support; (4) ion reservoir, which permit long-time electrochemical measurements; and (5) spacer, which allows the incorporation of bulky membrane proteins [9].

Up to now, most studies with SsLMs deal with the functional characterization of reconstituted membrane-active peptides and membrane proteins. In turn, the latter may act as sensing element if a ligand or other molecule interacts specifically with them and cause modification of the ion flow through the respective membrane-active peptide and membrane protein. 

The membrane-active peptide alamethicin forms channels in lipid membranes as well as in SsLMs on solid supports. Amiloride, an acid-sensing ion channel blocker inhibits these channels in a concentration dependent manner [122]. Increasing amounts of the ion channel blocker gave rise to a significant increase in membrane resistance as determined by EIS [71]. In turn, alamethicin channels reconstituted in SsLMs constitute sensing elements to determine the amiloride concentration in the surrounding aqueous milieu. Thus, this example shows proof of concept for the applicability of these composite S-layer/lipid structures for biosensing purposes. 

SsLMs, generated by the europium-induced vesicle fusion technique [123] provided a proper matrix for the insertion of gramicidin [69] and the voltage-dependent anion channel (VDAC) [70]. EIS studies probed the function of both, the membrane-active peptide gramicidin and the membrane protein VDAC. The incorporated VDAC caused a decrease in membrane resistance but the membrane capacitance did not vary significantly [70]. Moreover, the membrane resistance dropped down with increasing VDAC concentration indicating that an increasing number of functional channels spontaneously reconstituted into the SsLM [70]. VDAC is a voltage-gated channel, which is in the open state at a low membrane potential (˂ 10 mV) and switches to the closed state at high membrane potentials [124,125,126,127]. Indeed, the VDAC channels incorporated in SsLMs clearly showed this gating behavior. Furthermore, the membrane resistance decreased again after reducing the voltage from 10 mV back to zero. Moreover, the nucleotides nicotinamide adenine dinucleotide hydride (NADH) and nicotinamide adenine dinucleotide phosphate hydrogen (NADPH) are noted VDAC channel blockers [128,129,130,131]. Indeed, NADH caused a significant increase in SsLM resistance because of blocking the reconstituted VDAC channels [70]. Again, the VDAC channels reconstituted in SsLMs comprise a platform to determine the NADH and NADPH concentration in the surrounding aqueous milieu. Thus, the two before mentioned examples constitute proof-of-principle studies for the feasibility to utilize membrane-active peptides and membrane proteins reconstituted in SsLMs as electrochemical biosensor. The ability to reconstitute integral membrane proteins in defined structures like pure lipid bilayers [9,132,133,134,135], block copolymer bilayers [136,137], and recently lipid bilayers blended with block copolymers [138,139] on electrode and sensor surfaces is one of the most important concerns in designing biomimetic sensing devices in future [9,10,140,141,142].

A vigorous demand on membrane platforms like SsLMs is present because membrane proteins represent currently more than 50% of all drug targets [143,144]. Drug and membrane protein screening is; therefore, a vital evolving field in biosensor development because it bears the advantage of an amplification of the readout signal of membrane functions without need for labelling [105,145].

## 6. Conclusions and Outlook

Exploiting of bioinspired materials provides great contributions to biosensing applications and nanotechnology in general [146]. In the recent decades, nanotechnology-based sensing techniques for either analyte detection or biomimetic approaches have achieved noticeable success. Electrochemical biosensors are simple, accurate, and inexpensive tools. Moreover, decoration with bioinspired biomaterials to generate biosensing platforms with good selectivity and sensitivity for, for example, POC technology can easily done in particular if the biomolecules show the ability to self-assemble.

This review focuses on the exploitation of S-layer proteins in the development of electrochemical biosensors. The S-layer protein lattice constitutes a very promising intermediate layer because of its intrinsic chemical and physical characteristics (Table 1). Examples where S-layer protein-coated sensor surfaces provide a key function range from the detection of metal ions over the immobilization of enzymes to determine their function themselves or the conversion of a substrate by the enzyme to measuring specific receptor–ligand interactions of cells (Table 2). Moreover, it is even possible to discriminate between dead and alive cells. S-layer protein lattices provide also a unique scaffolding for supported lipid membranes. The retained fluidity of the latter allows the incorporation of membrane-active peptides and the reconstitution of membrane proteins like (G-protein coupled) receptors, ion channels, membrane-associated enzymes, and solute carries and transporters, which constitute nowadays the most important drug targets [83]. All these membrane-active biomolecules constitute highly sensitive and selective sensing elements for biosensor development and high-throughput screening devices in the field of diagnostic and drug discovery.

A challenge in the future is the integration of relevant key enabling technologies on an industrial scale (microfluidics with microelectronics, highly sensitive detection methods and low-cost materials for easy-to-use tools) [147,148,149,150] with biological components like S-layer proteins [7,10]. In general, biomolecules with self-assembly capability will play a pivotal role. This is not only because of miniaturization based on this bottom-up approach is rather easy to achieve, but also because of their molecular dimensions and repetitive features down to the nanoscale. In future, the S-layer lattice may constitute the key component for the design of smart biosensors relaying on two or more read-out techniques (e.g., electrochemical, microscopic, spectroscopic, acoustic, etc.), which will enable the determination of multiple parameters simultaneously.

The manufacturers of smart biosensors, lab-on-a-chip, and microfluidic devices will have to consider biomaterials as self-assembly components to realize important objectives in biosensor development like simple but reliable read-out system, volume reduction, high efficiency, low-cost, and easy-to-use. Finally, the integration in daily life and convenient handling for customers and patients will be the key parameter for the success of any diagnosis, POC diagnostics or other biosensor device. In this context, wearable biosensors are gaining vital interest due to their potential to provide continuous, real-time physiological information via noninvasive measurements of biochemical markers in biofluids, such as sweat, tears, saliva, and interstitial fluid [151].

## Figures and Tables

**Table 1:** Summary of the biosensor-relevant properties of S-layer proteins and lattices Reference.

Molecular weight of S-layer protein subunits: 40–200,000 Da	[7,23,24,25,26,27,28]
Reactive groups (e.g., carboxyl- and amino-residues) occur on each protomer in identical position and orientation	[7,23,24]
Two-dimensional (glyco)protein crystal composed of identical subunits	[7,25]
Oblique (p2), square (p4) or hexagonal (p6) space group symmetry	[7,16,26,27]
Center-to-center spacing of unit cells (= morphological units) of crystalline lattice: 3.5–35 nm	[7,27]
Layer thickness: 5–10 nm	[7,8]
High porosity (30%-70%) with pores of identical size (2–8 nm), morphology, and physicochemical properties	[7,24]
Topography: Inner surface smooth, outer surface more corrugated	[7,8,26]
Anisotropic charge distribution between outer and inner face: Outer face charge neutral due to an equal number of carboxyl- and amino groups. Inner face net negatively charged due to an excess of carboxyl groups	[7,8,24]
Antifouling, non-sticky outer surface	[7,11,28]
Self-assembly capability in aqueous media, on the air/water interface, on lipid films, and on solid surfaces like metals (gold, silver, platinum, stainless steel), glass, silicon, silicon oxide and nitride, mica, polymers (e.g., polystyrene, polyester, cellulose, polydimethylsiloxane (PDMS), indium tin oxide (ITO), highly oriented pyrolytic graphite (HOPG), and carbon nanotubes	[7,9,28,29,30]

**Table 2:** Summary of electrochemical biosensors based on S-layer proteins.

Electrode Architecture	Immobilization Method	Biorecognition Element	Detected Species	Electro-Chemical Method	Tested Detection Range	Linear Range	Stability	Remark	Reference
Au-SAM- SLP	Maleimide-Cys; biotin-avidin	SLP	UO_2_^2+^	EIS	10^−5^–10^−12^ M	10^−5^–10^−8^ M	N.D.	LOD 10^−12^ M	[91]
Au/SLP-GOx	Chemical (EDC)	GOx + FCN	glucose	Amperometric	0.5–50 mM	0.5–50 mM	2.2 h	blood, HSA, plasma	[28]
Au/Pt-GOx-SUM	Chemical (EDC)	GOx	glucose	Amperometric	2–20 mM	Up to 12 mM	48 h	Response 10–30 s	[62]
Au-AlcOx-SUM	Chemical (EDC)	alcohol oxidase	ethanol	Amperometric	-	Up to 7 mM	N.D.	Signal: 2.5 µA cm^−^^2^mM^−1^	[63]
Au-XanOx-SUM	Chemical (EDC)	xanthine oxidase	xanthine	Amperometric	-	Up to 0.6 mM	N.D.	Signal: 30 µA cm^−^^2^mM^−1^	[64]
Au-Maltase/ GOx-SUM	Chemical (EDC)	maltase + GOx	maltose	Amperometric	-	Up to 1.5 mM	N.D.	Signal: 1.5 µA cm^−^^2^mM^−1^	[64]
Au-Inv/Mut /GOx-SUM	Chemical (EDC)	invertase + mutarotase + GOx	sucrose	Amperometric	1–35 mM	Up to 30 mM	36 h	Response 300 s	[92]
C-ChOx/ SLP	Mixed multi-layers	ChOx	cholesterol	CV	3.1 mM	N.D.	N.D.	Langmuir/ Blodgett	[93]
Au-SLP/ZZ-anti-Ab	ZZ-domain + anti-CD133	anti-CD133 antibody	Liver cancer cells (HepG2)	CV	1 × 10^5^–6 × 10^6^ cells	Up to 6x10^6^ cells	N.D.	S-layer fusion protein	[72]
Au-SLP-folate	Chemical (EDC)	folate	Breast cancer cells (MFC-7)	SWV	1 × 10^4^–5 × 10^5^ cells	N.D.	N.D.	LOD 1 × 10^5^ cells/mL	[73]

Ab—antibody; Au—gold; Cys—cysteine; EDC-1-ethyl-3—(3-dimethylaminopropyl)carbodiimide; FCN—ferrocyanide/ferricyanide; HAS—human serum albumin; LOD—limit of detection; N.D. —not detected; SAM—self-assembled monolayer; SLP—S-layer protein; SUM—S-layer ultrafiltration membrane; ZZ—Fc-binding Z-domain, a synthetic analog of immunoglobulin G (IgG-binding B—domain) of protein A of *Staphylococcus aureus*.
